# Nanoarchitectonics of Carbon Nanostructures: Sodium Dodecyl Sulfonate @ Sodium Chloride System

**DOI:** 10.3390/nano12101652

**Published:** 2022-05-12

**Authors:** Qi Chen, Haichao Li

**Affiliations:** Key Laboratory of Applied Physical Chemistry of Qinghai Province, Qinghai Nationalities University, Xining 810007, China; chenqi19961114@163.com

**Keywords:** carbon nanostructures, surfactant, ordered aggregate, carbonization

## Abstract

Carbon nanostructures (carbon nanotubes, nano carbon spheres, layered carbon nanostructures) were prepared from a sodium dodecyl sulfonate @ sodium chloride system. Sodium dodecyl sulfonate was used as a carbon source. A sodium chloride crystal in the carbonization procedure was used to separate ordered aggregates of sodium dodecyl sulfonate. The results show that different carbon nanostructures were prepared at low concentrations (1CMC~5CMC) by controlling the concentration of sodium dodecyl sulfonate, such as nano carbon spheres and carbon nanotubes, and that layered carbon nanostructures were formed at high concentrations (10CMC). The prepared carbon nanostructures were characterized by transmission electron microscopy, fluorescence spectrometry and Raman spectrometry. The results of this experiment show that the surfactant @ salt system is a potential method for the preparation of carbon nanostructures.

## 1. Introduction

Since the advent of carbon nanostructures in the 1970s, although it has a history of more than 50 years, the research enthusiasm has never been reduced. Common carbon nanostructures include carbon nanotubes [1], carbon nanoangles [2], carbon nanodots [3], graphene [4] and other zero-dimensional, one-dimensional, two-dimensional and three-dimensional materials. The most widely studied carbon-based nanomaterials are carbon nanotubes and graphene. Carbon nanotubes are circular tubes with a spiral structure, and they are artificial synthetic carbon materials with the highest hardness and the best strength [5]. Graphene is a carbon-based material that is tightly stacked by single-layer carbon atoms in an SP^2^ hybrid orbit into a two-dimensional honeycomb structure [6]; graphene is expected to make possible not only the improvement of existing technologies, but also the development of new technologies unthinkable [7]. At present, the preparation of carbon nanostructures usually adopts the chemical vapor deposition method [8], oxidation–reduction method, combustion method, arc discharge method, pulse laser ablation method, electrospinning method and detonation synthesis method. However, these methods have certain defects such as a low yield of carbon nanostructures, harsh reaction conditions and high requirements for catalysts, which will inevitably lead to complex production processes and higher costs. Based on these, a new method for preparing carbon nanostructures with convenient operation and a low cost is proposed in this study.

**Figure 1:**
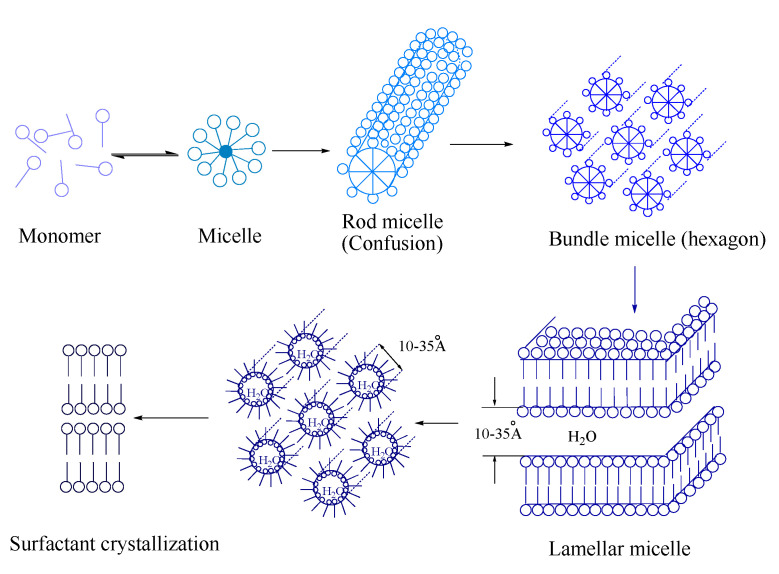
Structure formation in surfactant solution.

The electron arrangement of the carbon ground state is 1S^2^, 2S^2^, 2P^2^, and carbon nanostructures are orderly arranged by the hybridization of different hybrid orbitals [9], so carbon nanostructures can be obtained by the carbonization of ordered aggregates. A surfactant is a type of amphiphilic molecule that is both lipophilic and hydrophilic; this molecule will adopt a unique directional arrangement compared to a water medium in an aqueous solution system (including the surface and interface) and form a certain structural organization. Sodium dodecyl sulfonate (SDSN) is an anionic surfactant, and Hartley [10] proposed that the micelles are spherical and have a certain size in CMC aqueous media. With the increase in the solution concentration, the aggregation shape of the micelles changes to obtain various structures (Figure 1). In primary experiments, if the SDSN solution is carbonized directly, carbon nanostructures will not be obtained because of agglomeration. In order to solve this problem, a certain salt was added to the solution to separate the SDSN micelles in the solution. Since sodium chloride (NaCl) is resistant to high temperature and belongs to neutral salts, the surfactant micelles should not be greatly affected. After stirring and evaporation, the SDSN @ NaCl system was formed and carbonized. In the carbonization process, NaCl plays a role in separation and also in air isolation, so that the carbonization process does not require the protection of rare gases. It can be carbonized directly in a muffle furnace connected with the air. Finally, carbon nanostructures with different morphologies were successfully obtained. Cleaner production can be achieved by drying the filtrate after washing the carbonization products to recover NaCl. Compared with other methods, this method has the advantages of convenient operation, a low cost and no need for a catalyst, and it also has strong practical application ability.

## 2. Experimental

### 2.1. Preparation of SDSN Solution

The CMC value of SDSN in a water medium at 40 °C was 9.7 × 10^−3^mol/L. SDSN (analytical reagent, 99.98%, Aladdin, Shanghai, China) at 1, 2, 5 and 10 times the CMC was dissolved in 100 mL water using an electronic balance and a volumetric flask.

### 2.2. Preparation of SDSN @ NaCl System

The SDSN @ NaCl was grown using the evaporative crystallization method. The prepared solution was placed in a 200 mL beaker, and the beaker was reposed on a magnetic heating stirrer to add a magnet. The temperature of the magnetic heating stirrer was adjusted to 200 °C, and the rotation speed was 300 r/min. When the solution was clear, it showed that SDSN formed the corresponding micelles, and NaCl (analytical reagent, 98.98%, Aladdin, Shanghai, China) was gradually added to the solution until crystallization occurred in the solution. The SDSN @ NaCl system was successfully prepared by continuing stirring and heating to evaporate all the water in the SDSN solution to form a white blending solid.

### 2.3. Preparation of Carbon Nanostructures

**Figure 2:**
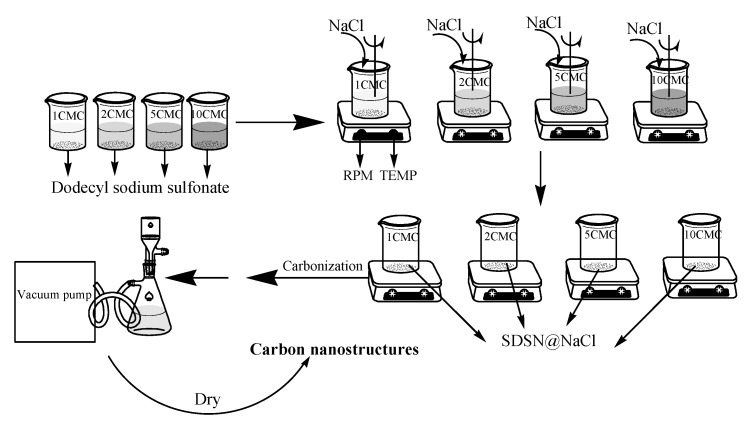
Experimental process diagram.

The prepared SDSN @ NaCl was placed in a crucible and reposed in a box-type resistance furnace. The temperature was raised to 500 °C, 600 °C and 800 °C at a rate of 5 °C/min. The samples were kept at the corresponding temperature for 1h to ensure that they were fully carbonized. The box-type resistance furnace was closed to wait for automatic cooling to room temperature to remove the samples, and then the carbonized products of SDSN @ NaCl at various temperatures were obtained (Figure 2).

### 2.4. After-Treatment

The carbonized products extracted from the box-type resistance furnace were grinded to a powder by a mortar and then added into a 1000 mL beaker to soak in ultrapure water for 24 h to make the carbon nanostructures aggregate. The carbonized products were filtered by a vacuum pump and a sand core filter and dried at 60 °C for 5~10 h in a constant temperature drying oven.

### 2.5. Characterization

The carbon nanostructures were characterized by transmission electron microscopy (TEM) (FEI tecnai G2 F30, USA), fluorescence spectrometry (PL) (Hitachi, F-400, Japan) and Raman spectrometry (Raman) (Horiba HR Evolution, France).

Transmission electron microscopy was used to observe the microstructure information of the carbon nanostructures and understand their morphology under different concentrations of SDSN. Samples were prepared for transmission electron microscopy (TEM) by dispersing some of the as-prepared products in ethanol with sonication and dropping a small amount of the dispersed product onto carbon-coated grids [11]. The prepared spherical carbon nanostructures were characterized by fluorescence spectroscopy. The samples were excited at 360 nm, 380 nm, 400 nm and 420 nm to obtain PL spectra. Additionally, the sample was excited by 532 nm visible light to obtain its Raman spectrum. [12].

## 3. Results and Discussion

### 3.1. TEM Image Analysis of Carbon Nanostructures

#### 3.1.1. TEM Images of Carbon Nanostructures at 500 °C

**Figure 3:**
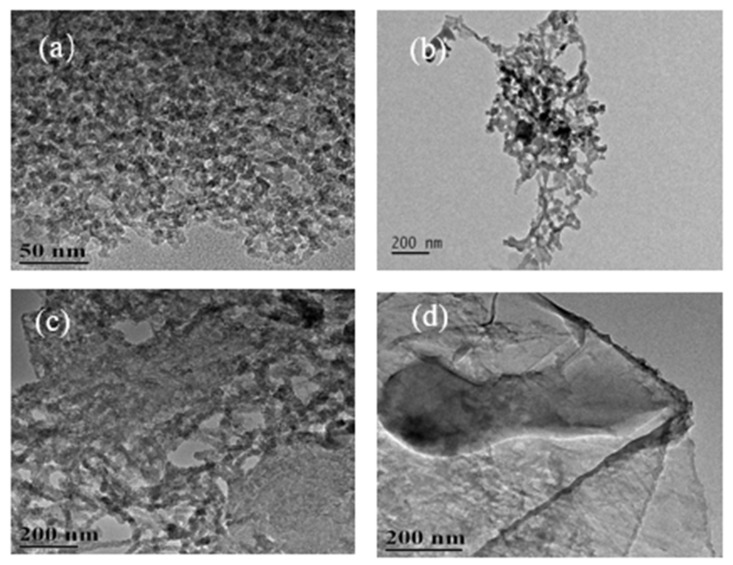
TEM images of carbon nanostructures at 500 °C: (**a**) 1CMC concentration carbon nanostructures; (**b**) 2CMC concentration carbon nanostructures; (**c**) 5CMC concentration carbon nanostructures; (**d**) 10CMC concentration carbon nanostructures.

The TEM images of the carbon nanostructures obtained by the carbonization of different concentrations of SDSN @ NaCl at 500 °C are shown in Figure 3 [13]. Figure 3a shows the formation of spherical carbon nanostructures at the concentration of 1CMC. With the concentration reaching 2CMC (Figure 3b), the particle size of the spherical carbon nanostructures increased, and the agglomeration phenomenon increased. When the concentration increased to 5CMC (Figure 3c), the morphology of the carbon nanostructures changed into a tubular structure. Figure 3d shows that when the concentration reached 10CMC, layered micelles formed due to SDSN, so the layered carbon nanostructures were formed after carbonization.

#### 3.1.2. TEM Images of Carbon Nanostructures at 600 °C

**Figure 4:**
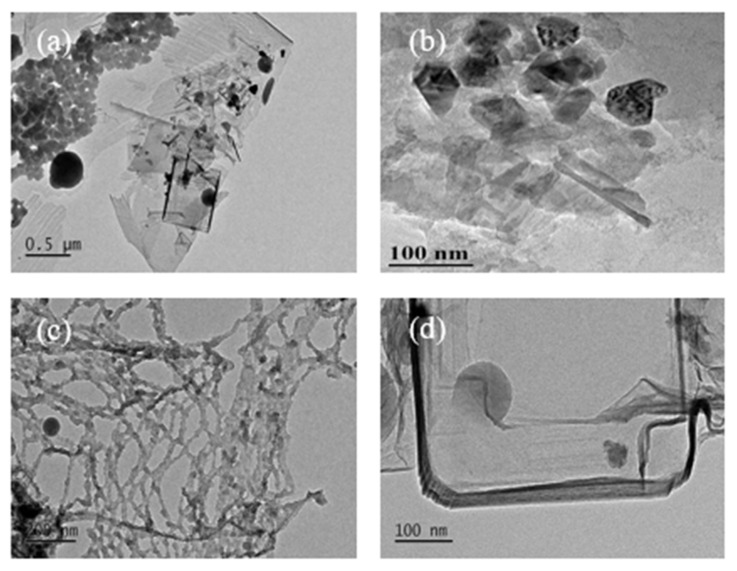
TEM images of carbon nanostructures at 600 °C: (**a**) 1CMC concentration carbon nanostructures; (**b**) 2CMC concentration carbon nanostructures; (**c**) 5CMC concentration carbon nanostructures; (**d**) 10CMC concentration carbon nanostructures.

Carbon nanostructures were obtained by the carbonization of the SDSN @ NaCl system at 600 °C, as shown in Figure 4. The TEM images of carbon nanostructures formed at four concentrations of a, b, c and d were not significantly different from those at 500 °C. The difference was that when the carbonization temperature was 600 °C, the carbonization effect was better due to the increase of temperature, and the formation of carbon nanostructures were more complete and clearer. 

#### 3.1.3. TEM Images of Carbon Nanostructures at 800 °C

**Figure 5:**
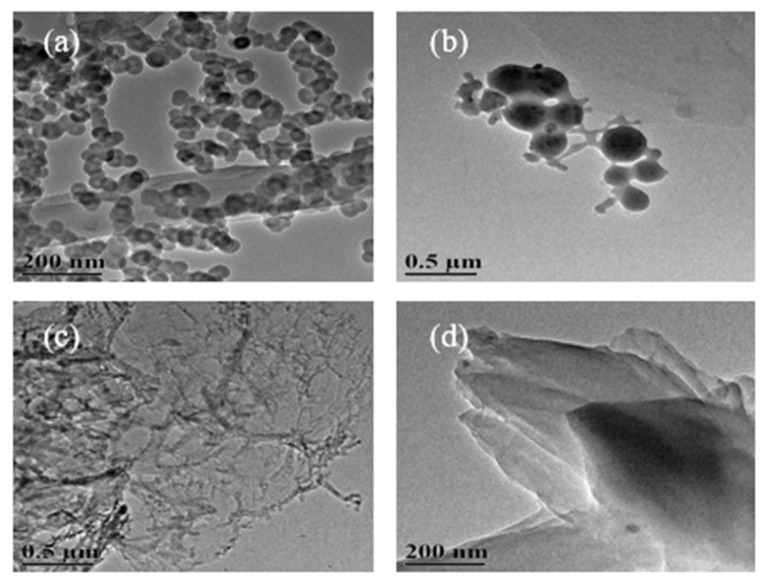
TEM images of carbon nanostructures at 800 °C: (**a**) 1CMC concentration carbon nanostructures; (**b**) 2CMC concentration carbon nanostructures; (**c**) 5CMC concentration carbon nanostructures; (**d**) 10CMC concentration carbon nanostructures.

Similarly, the carbon nanostructures obtained by the carbonization of the SDSN @ NaCl system at 800 °C are shown in Figure 5. Compared with Figure 3, the morphology of the obtained carbon nanostructures is similar, but compared with Figure 4, the morphology of carbon nanostructures is not more clear and complete, and serious agglomeration occurs. This is due to the hysteresis phenomenon of the muffle furnace when the carbonization temperature is set at 800 °C, which leads to the temperature exceeding the melting point of NaCl (801 °C), as a result, NaCl does not play a better role in separating carbonization, resulting in serious agglomeration of the carbon nanomaterials.

### 3.2. PL Spectrum Analysis of Spherical Carbon Nanostructures

**Figure 6:**
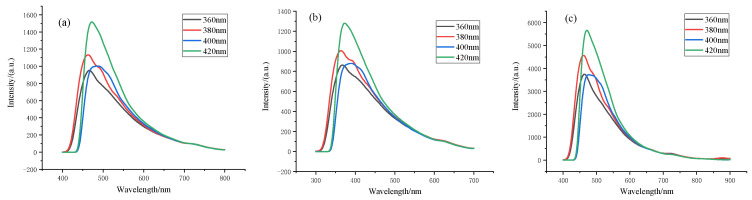
Fluorescence spectra of spherical carbon nanostructures at three temperatures: (**a**) spherical carbon nanostructures at 500 °C; (**b**) spherical carbon nanostructures at 600 °C; (**c**) spherical carbon nanostructures at 800 °C.

The spherical carbon nanostructures prepared at three temperatures were excited by a wavelength of 360 nm–420 nm with an interval of 20 nm. Figure 6 shows the PL spectra of the three spherical carbon nanostructures.

It can be seen from Figure 6 that the optimal excitation wavelength of the three spherical carbon nanostructures was 420 nm; when the excitation wavelength increased from 360 nm to 420 nm, the emission wavelengths of samples a and c were similarly concentrated between 430 nm and 700 nm, and the emission wavelength of sample b was concentrated between 330 nm and 600 nm. At the same time, with the increase in the excitation wavelength, the three spherical carbon nanostructures all had different degrees of red-shift, indicating that the fluorescence of the three spherical carbon nanostructures was closely related to the excitation wavelength, which may be the result of the surface defects of the spherical carbon nanostructures [14,15,16]. This indicates that the polar functional groups on the surface of the spherical carbon nanostructures reduced the energy level difference of the π−π* transition. Therefore, with the increase in surface chromophores, the PL spectrum of the spherical carbon nanostructures red-shifted [17]. The upconversion luminescence properties of the spherical carbon nanostructures have great application prospects in high-efficiency photocatalysis, photovoltaic devices, energy conversion and other fields [18].

### 3.3. Analysis of Graphitization Degree

**Figure 7:**
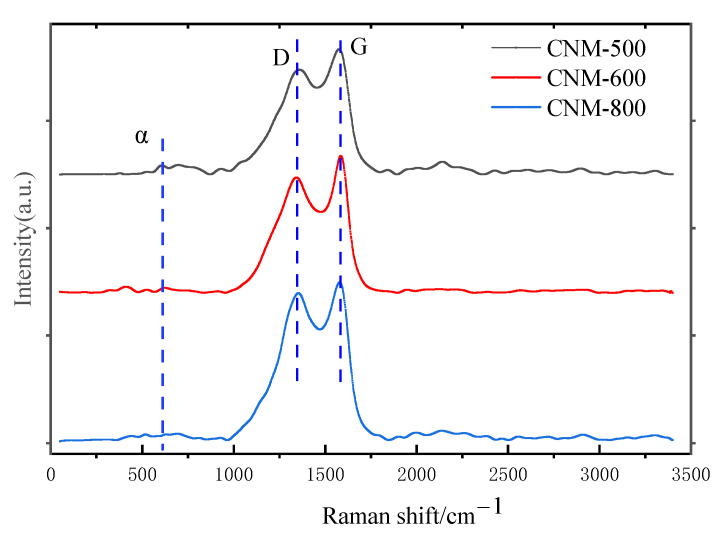
Raman spectra of carbon nanostructures at different temperatures.

Graphitization is usually understood as a phase transformation of C-C SP^3^ to C-C SP^2^ bonds in metastable diamonds [19]. Raman spectroscopy is a direct and non-destructive analysis method to characterize the structural properties of carbon nanostructures such as defects, disorder and doping. The Raman spectra of the carbon nanostructures obtained at the three temperatures are shown in Figure 7. The Raman spectra of the carbon nanostructures show the G and D bands that are characteristic for graphitic structures, where the G band (at ~1580 cm^−1^) originates from the ordered, well-graphitized carbon, while the D band (at ~1360 cm^−1^) is the disorder-activated band [20,21]. The ratio of the area of the D band to G band (*I_D_/I_G_* ratio) in Raman spectra has been used extensively as a measure of the graphitization of a sample, and as a measure of the quality of the carbon nanostructures produced: a smaller *I_D_/I_G_* ratio corresponds to fewer defects [22]. There is a characteristic peak α band at 600 cm^−1^, which is due to the oxygen vacancies in the carbonation process. With the increase in the experimental temperature, the oxygen vacancies decreased gradually [23].

The carbon nanomaterial *I_D_/I_G_* ratios obtained at the three temperatures were 2.973, 2.731 and 2.812. The *I_D_/I_G_* value at 600 °C was the smallest, indicating that the carbon nanostructures at 600 °C had the least defects and a more complete crystal structure; when the temperature was 500 °C, the degree of graphitization was the lowest; when the temperature was 800 °C, NaCl melted and covered the surface of SDSN, resulting in the carbonization temperature not reaching the set temperature.

## 4. Conclusions

The synthesis of carbon nanostructures was carried out by a new method. Ordered aggregates can be obtained by controlling the surfactant concentration. Ordered aggregates have spherical, tubular, layered, wormlike shapes. The direct carbonization of ordered aggregates will destroy the ordered structure. In this study, the ordered aggregates were evenly separated by salt crystallization, and on this basis, carbon nanostructures with different shapes were obtained by carbonization. Nano carbon spheres were formed at the concentration of SDSN1CMC, carbon nanotubes were formed at the concentration of SDSN5CMC and layered carbon nanostructures were formed at the concentration of SDSN10CMC. This method is based on the specific structure of the surfactant in the aqueous solution and the idea of salt wrapping, effectively avoiding the current preparation of carbon nanostructures which entails a complex process, harsh reaction conditions, a high cost and many other defects. In this paper, the best carbonization temperature was 600 °C under the three temperatures studied. The prepared carbon nanostructures had the highest graphitization degree and a pure phase. At the same time, this method can be extended to other carbonized surfactants and high-temperature salt systems. It has a strong application prospect to prepare different carbon nanostructures by controlling the concentration of surfactants.

## Data Availability

Not applicable.
